# Interplay Between Mitophagy and Apoptosis Defines a Cell Fate Upon Co-treatment of Breast Cancer Cells With a Recombinant Fragment of Human κ-Casein and Tumor Necrosis Factor-Related Apoptosis-Inducing Ligand

**DOI:** 10.3389/fcell.2020.617762

**Published:** 2021-01-18

**Authors:** Fabian Wohlfromm, Max Richter, Lado Otrin, Kamil Seyrek, Tanja Vidaković-Koch, Elena Kuligina, Vladimir Richter, Olga Koval, Inna N. Lavrik

**Affiliations:** ^1^Translational Inflammation Research, Medical Faculty, Otto von Guericke University, Magdeburg, Germany; ^2^Max Planck Institute for Dynamics of Complex Technical Systems, Magdeburg, Germany; ^3^Department of Biotechnology, Institute of Chemical Biology and Fundamental Medicine, Siberian Branch of Russian Academy of Sciences (SB RAS), Novosibirsk, Russia

**Keywords:** apoptosis, mitophagy, RL2, TOM70, TRAIL, lactaptin, milk proteins and peptides

## Abstract

A recombinant fragment of human κ-Casein, termed RL2, induces cell death of breast cancer cells; however, molecular mechanisms of RL2-mediated cell death have remained largely unknown. In the current study, we have decoded the molecular mechanism of the RL2-mediated cell death and found that RL2 acts via the induction of mitophagy. This was monitored by the loss of adenosine triphosphate production, LC3B-II generation, and upregulation of BNIP3 and BNIP3L/NIX, as well as phosphatase and tensin homolog-induced kinase 1. Moreover, we have analyzed the cross talk of this pathway with tumor necrosis factor-related apoptosis-inducing ligand (TRAIL)-induced apoptosis upon combinatorial treatment with RL2 and TRAIL. Strikingly, we found two opposite effects of this co-treatment. RL2 had inhibitory effects on TRAIL-induced cell death upon short-term co-stimulation. In particular, RL2 treatment blocked TRAIL-mediated caspase activation, cell viability loss, and apoptosis, which was mediated via the downregulation of the core proapoptotic regulators. Contrary to short-term co-treatment, upon long-term co-stimulation, RL2 sensitized the cells toward TRAIL-induced cell death; the latter observation provides the basis for the development of therapeutic approaches in breast cancer cells. Collectively, our findings have important implications for cancer therapy and reveal the molecular switches of the cross talk between RL2-induced mitophagy and TRAIL-mediated apoptosis.

## Introduction

Apoptosis is a program of cell death that is essential for all multicellular organisms (Krammer et al., 2007; Lavrik and Krammer, 2012). Its deregulation is associated with several diseases, including cancer. Several programs of cell death have been discovered to date (Galluzzi et al., 2018). Cross talk between different cell death modalities plays a key role in shaping life/death decisions in the cell. Furthermore, the success of anticancer therapies strongly depends on the efficiency of cell death induction and intrinsic cross talk of several cell death modalities. However, it is well known that many combinatorial treatments can also induce strong antiapoptotic responses. This can prevent apoptosis by upregulation of antiapoptotic genes and, hence, counteract the effect of anticancer therapies (Buchbinder et al., 2018). Accordingly, the detailed analysis of the cell death network upon administration of several cell death stimuli plays a key role in the development of contemporary anticancer therapies.

Tumor necrosis factor-related apoptosis-inducing ligand (TRAIL)-based agents are promising anticancer therapeutics that are in clinical trials for several cancer therapies, including breast cancer (Lemke et al., 2014; von Karstedt et al., 2017). TRAIL is a member of the death ligand family (Lafont et al., 2017). Binding of TRAIL to the specific receptors (TRAILR1/2) leads to the formation of the death-inducing signaling complex (DISC) and initiation of the extrinsic apoptosis pathway as well as caspase activation (Sprick et al., 2000; Spencer et al., 2009; Lafont et al., 2017). TRAIL receptors 1 and 2 belong to the death receptor family and are also named death receptors 4 and 5. In addition to the receptors, the adaptor protein Fas-associated protein with death domain (FADD), the initiator procaspase-8a/b (p55/p53), procaspase-10, and cellular FADD-like interleukin-1β-converting enzyme-inhibitory protein (c-FLIP) are part of the DISC (Sprick et al., 2000; Walczak and Haas, 2008). After recruitment to the DISC, procaspase-8a/b builds death effector domain (DED) filaments, formed via homotypic interactions between the DEDs of individual procaspase-8 molecules (Dickens et al., 2012; Schleich et al., 2012; Fu et al., 2016). This provides the platform for homodimerization of procaspase-8 molecules, its subsequent activation and processing of procaspase-8a/b to p43/p41, p30, and formation of caspase-8 heterotetramers p10_2_-p18_2_ (Lavrik et al., 2003; Hoffmann et al., 2009; Dickens et al., 2012). The activation of procaspase-8 is blocked by c-FLIP proteins (Hughes et al., 2016; Hillert et al., 2020).

There are two types of TRAIL signaling downstream of the DISC, which take place in the so-called type I and type II cells (Aldridge et al., 2011; von Karstedt et al., 2017). In type I cells, a high amount of the TRAIL DISC is formed, followed by the generation of high amounts of caspase-8 and subsequent cell death. In type II cells, smaller quantities of caspase-8 are formed at the DISC, and, accordingly, the propagation of cell death requires the mitochondrial amplification loop, which is mediated via Bid cleavage to tBid, translocation of tBid to mitochondria, cytochrome C release from the mitochondria, and caspase-9 activation (von Karstedt et al., 2017). Apoptosis induction in type II cells is controlled by X-linked inhibitor of apoptosis protein/caspase-3 ratios and can be blocked by overexpression of Bcl-2/Bcl-XL (Scaffidi et al., 1998; Aldridge et al., 2011; Kaufmann et al., 2012).

Lactaptin is the proteolytic fragment of the human milk protein κ-Casein (Semenov et al., 2010). A recombinant analog of lactaptin, the peptide RL2 (recombinant lactaptin 2), which comprises amino acid 23-134 of human κ-Casein, has been described to induce cell death of breast carcinoma cells (Semenov et al., 2010). In particular, this peptide was shown to induce cell death in MDA-MB-231 and MCF-7 cells and suppress tumor growth in mice models (Koval et al., 2012, 2014). RL2 was reported to modulate the expression of apoptotic proteins and induce autophagy in MDA-MB-231 cells (Koval et al., 2014; Bagamanshina et al., 2019). RL2 acts in monomeric, dimeric, and oligomeric forms; the latter are formed via cysteine bridges (Chinak et al., 2019). Moreover, recently, it has been shown that RL2 mediates cell death, which was accompanied by the loss of mitochondrial membrane potential and intracellular adenosine triphosphate (ATP) loss (Richter et al., 2020). Mass spectrometry-based screening of RL2 interactome identified the mitochondrial import protein TOM70 as an interaction partner of RL2. Further, it was unveiled that RL2 is targeted to the mitochondria after internalization into the cells (Richter et al., 2020). The requirement for TOM70/RL2 interaction in RL2-induced reduction of intracellular ATP levels was validated by downregulation of TOM70, resulting in partial rescue of the intracellular ATP yield. Taken together, it was shown that RL2-mediated cell death is mediated via mitochondria, in particular via interactions with TOM70. However, the detailed mechanisms of RL2 action at mitochondria leading to cell death needs to be further investigated. In particular, it remains unknown which molecular pathway plays a major role in RL2-mediated ATP loss and RL2-induced cell death.

In the current study, we uncover that RL2 acts via induction of mitophagy, leading to the ATP down-modulation. Moreover, we have analyzed the cross talk of this pathway with TRAIL-induced apoptosis and the effects of RL2 on TRAIL-induced apoptotic signaling. To this end, we addressed the interplay of extrinsic apoptosis and mitophagy upon co-administration of TRAIL and RL2 in breast cancer cells and delineated the major molecular switches between these pathways. Both agents are considered potent cell death inducers in breast cancer cells, and therefore, their combination is highly promising due to its putative therapeutic applications. Therefore, the current study leading to the understanding of the molecular mechanism of RL2/TRAIL co-treatment plays an important role in developing new therapeutic approaches for breast cancer.

## Results

### RL2 Blocks Tumor Necrosis Factor-Related Apoptosis-Inducing Ligand-Induced Cell Viability Loss in Breast Cancer Cells

**Figure 1:**
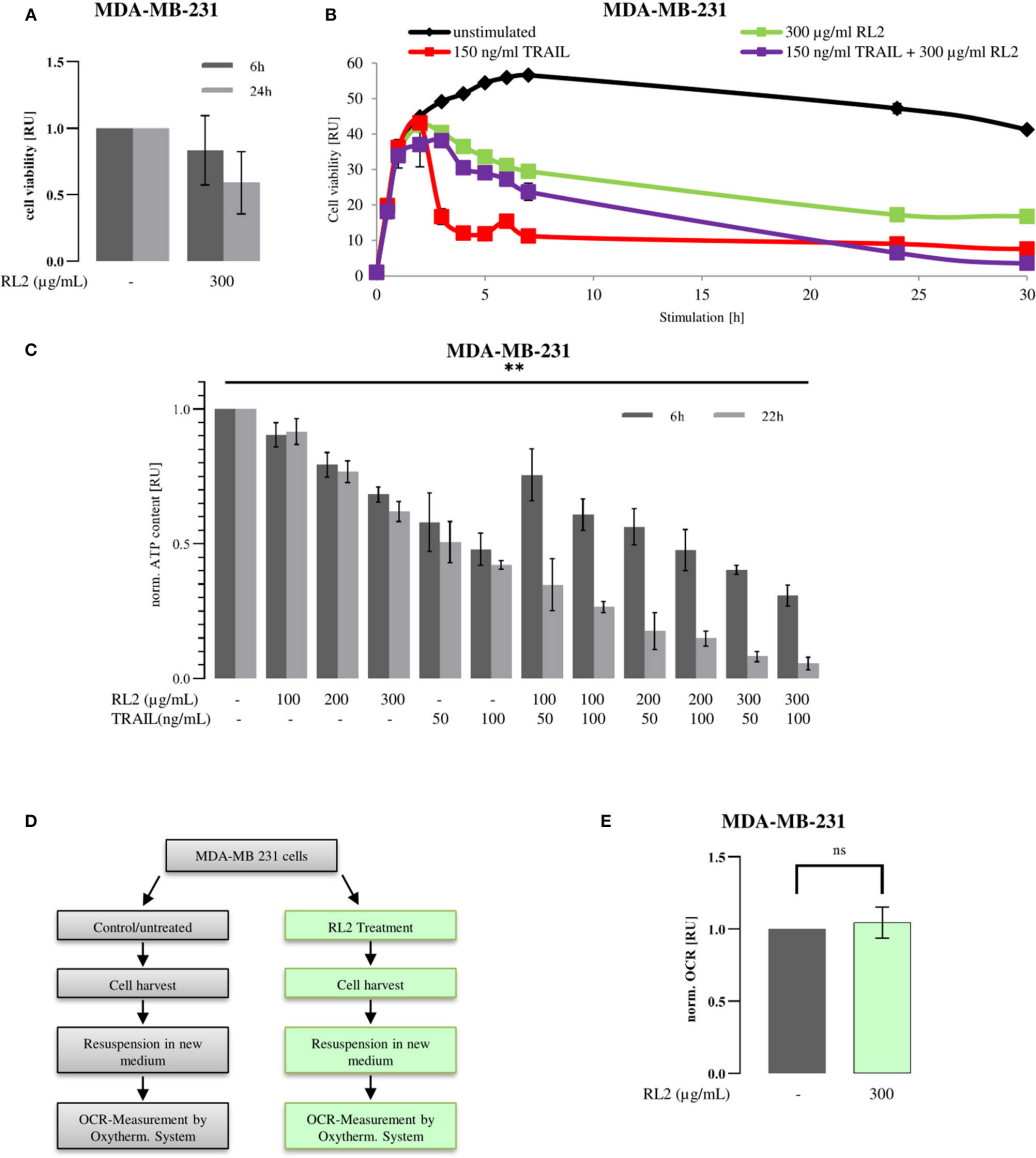
RL2 inhibits TRAIL-induced cell viability loss in MDA-MB-231 cells during the first hours of TRAIL stimulation. MDA-MB-231 cells were treated with indicated concentrations of RL2, TRAIL, or their combination for 6–48 h. **(A,B)** Cell viability was measured using the metabolic RealTime-Glo MT Cell Viability Assay. **(C)** Cellular ATP levels were measured using CellTiter-Glo Luminescent Cell Viability Assay/CellTiter-Glo Substrate, and cell viabilities are normalized to the ones of non-treated cells and presented in relative units. Mean and standard deviations are shown (*n* = 3). Statistical analysis was performed for 6 and 22 h by ANOVA test **(C)**. **(D)** Workflow for oxygen consumption rate (OCR) measurement after RL2 treatment. Cells were treated (green) or remained untreated (gray) for 8 h. Then, medium was aspirated, and cells were harvested. Cells were resuspended in fresh media, and OCR was measured by Oxytherm System (Hansatech Instruments Ltd, Norfolk, UK). **(E)** OCR measurements on RL2-treated MDA-MB-231 cells. Mean and standard deviations are shown (*n* = 3). Statistical analysis was performed by Student's *t*-test.

RL2 treatment alone induces cell viability loss of breast cancer cells (Figure 1A), whereas TRAIL treatment alone causes the cell death of TRAIL-sensitive cells (Sprick et al., 2000; von Karstedt et al., 2017; Richter et al., 2020). To analyze the effects of RL2/TRAIL coadministration on cell viability, the treatment of MDA-MB-231 breast carcinoma cells with RL2, TRAIL, or their combination was carried out (Figure 1B). RL2 treatment alone caused only a slight cell viability loss, which was measured by metabolic assays, in the 6 h after its administration (Figures 1A,B). This was in line with previous reports (Richter et al., 2020). TRAIL addition alone led to a more pronounced cell viability loss compared with RL2. Already 4 h after TRAIL-only administration, the cell viability was strongly down-modulated compared with RL2-only treatment (Figure 1B). Strikingly, the coadministration of RL2 blocked TRAIL-mediated cell viability loss upon short-term treatment. In particular, 4 h after RL2/TRAIL co-stimulation, only a slight reduction in cell viability loss was observed, which was in contrast to the strong TRAIL-induced cell viability loss observed during this time interval (Figure 1B). However, the inhibitory effects of RL2 disappeared after longer stimulation of MDA-MB-231 cells. Moreover, 24 h after stimulation, RL2/TRAIL-induced cell viability loss was stronger than that mediated by TRAIL only or RL2 only (Figure 1B).

RL2 treatment of breast carcinoma cells induces the intracellular ATP loss that accompanies RL2-mediated cell death (Richter et al., 2020). The ATP loss might be considered as an indirect indication of cell viability loss. Hence, next, we analyzed the effects of RL2/TRAIL co-treatment on MDA-MB-231 cells via measuring intracellular ATP content (Figure 1C). In these experiments, in accordance with previous reports, RL2 treatment alone led to ATP down-modulation (Richter et al., 2020). Similarly, TRAIL treatment alone also led to strong ATP loss. Moreover, similar to the effects observed in Figure 1B, RL2/TRAIL co-treatment led to the inhibitory action of RL2 on TRAIL-induced ATP loss in MDA-MB-231 cells upon short-term treatment (Figure 1C). Strikingly, similar inhibitory effects of RL2 on TRAIL-induced cell viability loss were also observed on the other cell lines, such as ovarian carcinoma CAOV-4 cells (Supplementary Figure 1). Furthermore, analogous to the observations made using metabolic assays on MDA-MB-231 cells (Figure 1B), the inhibitory effects of RL2 disappeared after longer stimulation intervals (Figure 1C). Taken together, these experiments suggest that TRAIL and RL2 co-treatment blocks short-term and promotes long-term TRAIL-induced cell viability and ATP loss. Next, we aimed at uncovering the mechanisms behind these observed effects.

Mitochondrial respiratory complexes drive ATP production. RL2 blocks intracellular ATP production (Figure 1C), which might occur via perturbing the function of the mitochondrial respiratory chain. However, it is known that cells with compromised respiration might shift energy production to anaerobic glycolysis. The latter might take place upon RL2 treatment of the cells. To test this hypothesis, the oxygen consumption rate of MDA-MB-231 cells upon RL2 treatment was measured using a Clark-type electrode (Figures 1D,E). Strikingly, no alterations in the oxygen consumption were detected upon RL2 treatment (Figure 1E). This allows us to conclude that RL2 action does not directly involve perturbation of the mitochondrial respiratory chain, and other mechanisms leading to the ATP loss upon RL2 treatment were further investigated.

### RL2 Blocks Tumor Necrosis Factor-Related Apoptosis-Inducing Ligand-Induced Caspase-8 Activation at the Death-Inducing Signaling Complex

**Figure 2:**
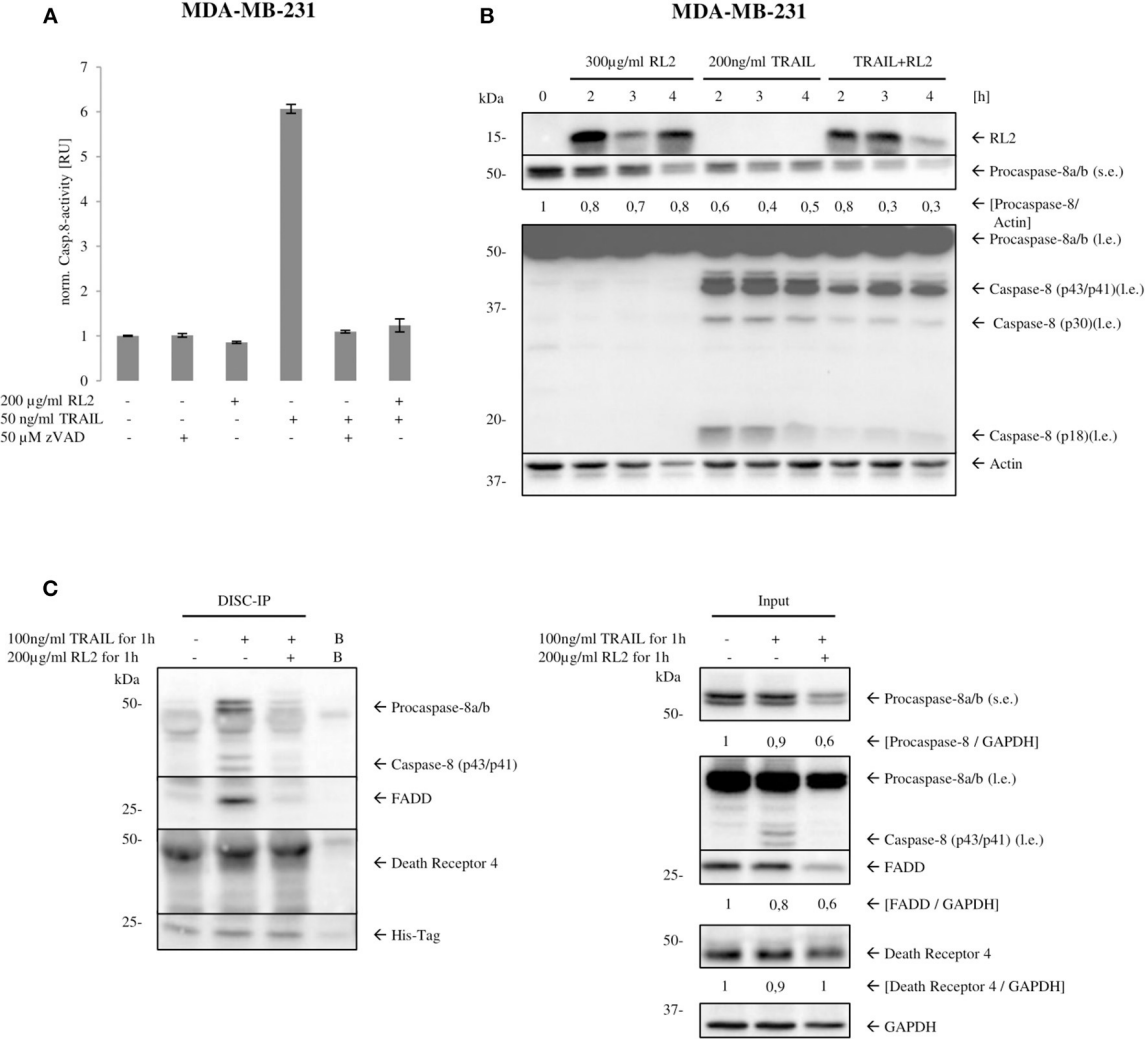
RL2 reduces caspase-8 activity at the DISC. **(A)** MDA-MB-231 cells were treated with 200 μg/ml RL2, 50 ng/ml of TRAIL, or their combination for 3 h. Samples, which were pretreated with 50-μM pan-caspase inhibitor zVAD-fmk for 1 h, were used as a negative control. Caspase-8 activity was determined using Caspase-Glo 8 Assay. Caspase activity is normalized against untreated sample, and activity is shown in relative units. One representative assay of three independent ones is shown. **(B)** MDA-MB-231 cells were treated with 300 μg/ml RL2, 200 ng/ml TRAIL, or their combination for indicated time points and subjected to Western blot analysis with indicated antibodies. Procaspase-8 cleavage products p43/p41, p30, and p18 are indicated. One representative Western blot of two independent experiments is shown. l.e., long exposure; s.e., short exposure. Protein expression was quantified and normalized to actin. Quantification is shown under the specific bands. **(C)** MDA-MB-231 cells were treated with the indicated TRAIL, RL2, and RL2/TRAIL concentrations for 1 h. Co-IPs were performed with 5-μg anti-6 x His-Tag antibody (DISC-IP). Lysate (input) and DISC-IP were analyzed by Western blot for the indicated proteins. “Bead control” **(B)**: IP without any antibody addition. One representative experiment of two is shown.

TRAIL treatment leads to induction of the extrinsic apoptosis pathway, which is orchestrated by caspase activation. Caspase-8 is an apical or initiator caspase of TRAIL-induced apoptosis, triggering the effector caspase cascade. To find out whether inhibition of effector caspases occurs already at the level of initiator caspase activation, caspase-8 activity in MDA-MB-231 cells upon RL2, TRAIL, or RL2/TRAIL treatment for 3 h was measured (Figure 2A). TRAIL-only treatment resulted in a strong induction of caspase-8 activity after TRAIL stimulation, which is in line with previous reports (Sprick et al., 2000, 2002). RL2 alone did not cause any increase in caspase-8 activity. Moreover, the addition of RL2 strongly blocked the induction of caspase-8 activity induced by TRAIL (Figure 2A). These findings were further supported by a Western blot analysis of procaspase-8a/b processing, which indicated that RL2 co-treatment strongly diminished TRAIL-induced procaspase-8a/b processing to p43/p41, p30, and p18 (Figure 2B). Taken together, these results indicate that RL2 inhibits TRAIL-induced caspase activation already at the level of initiator caspase-8.

Upon TRAIL stimulation, caspase-8 is activated at the TRAIL DISC, comprising TRAILR1/2, FADD, procaspase-8/10, and c-FLIP proteins (Sprick et al., 2000; von Karstedt et al., 2017). In addition, caspase-8 has been reported to be activated in complex IIa, which also comprises the core components of the DISC: FADD, procaspase-8/10, and c-FLIP proteins (Lafont et al., 2017; von Karstedt et al., 2017). Because caspase-8 activation was inhibited upon RL2/TRAIL co-treatment, the next step was to test whether RL2 is recruited to the macromolecular complexes formed upon TRAIL stimulation and thereby interferes with caspase-8 activation. To this point, caspase-8 and FADD co-immunoprecipitations (co-IPs) were carried out with MDA-MB-231 cells using anti-caspase-8 and anti-FADD antibodies, respectively. These co-IPs should pull-down both complex II and DISC. RL2 stimulation alone did not trigger the association of FADD, procaspase-8, and c-FLIP proteins (Supplementary Figures 2A,B). TRAIL-only stimulation led to the association of procaspase-8, FADD, and c-FLIP, which were detected in both FADD co-IP (Supplementary Figure 2A) and caspase-8 co-IP (Supplementary Figure 2B). However, no association of RL2 with the core TRAIL DISC components was observed upon RL2/TRAIL co-treatment, neither in the FADD co-IP (Supplementary Figure 2A) nor in the caspase-8 co-IP (Supplementary Figure 2B). Moreover, the levels of association between FADD, procaspase-8, and c-FLIP were not changed upon the addition of RL2. These results strongly indicate that RL2 is not directly associated with FADD, procaspase-8, or c-FLIP. However, these results do not exclude that the composition of the TRAIL DISC upon RL2 co-stimulation might be different, as co-IP via FADD and procaspase-8 might result in the different ratios of these proteins than in the native DISC complex.

To check this hypothesis, we performed DISC co-IP (Figure 2C). This co-IP was performed via the His-tagged TRAIL, which allowed to pull-down FADD, procaspase-8, and TRAILR upon TRAIL stimulation (Figure 2C). Indeed, in accordance with our suggestion, the ratios of these proteins were different at the DISC from the complex II. In particular, upon RL2/TRAIL co-treatment, decreased amounts of procaspase-8a/b and FADD at the TRAIL DISC were observed compared with TRAIL only treatment (Figure 2C). Furthermore, we have detected the downregulation of FADD and procaspase-8 in the corresponding cellular lysates upon RL2 treatment, whereas the amounts of TRAILR1 remained unchanged in similar conditions (Figure 2C). This allowed us to draw a conclusion that RL2 treatment leads to the decrease of procaspase-8 and FADD amounts, which results in the less efficient DISC formation and caspase-8 activation.

### RL2 Blocks Tumor Necrosis Factor-Related Apoptosis-Inducing Ligand-Induced Effector Caspase Activation

**Figure 3:**
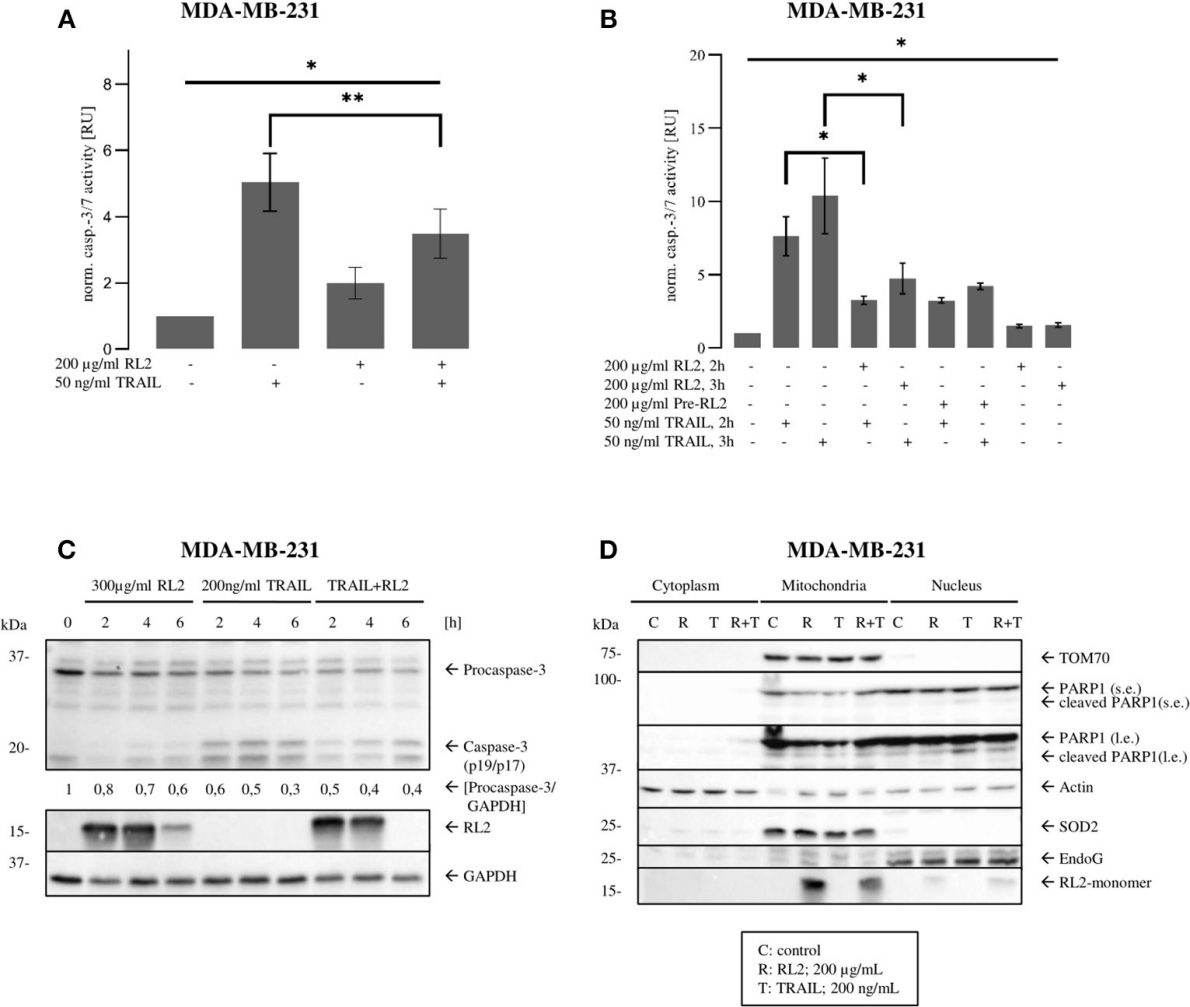
RL2 inhibits TRAIL-induced effector caspase activity in MDA-MB-231 cells during first hours after TRAIL stimulation. **(A,B)** MDA-MB-231 cells were treated with indicated concentrations of RL2, TRAIL, the combination of both, or 2-h pretreatment by RL2 for 3 h. Caspase-3/-7-activity was determined using Caspase-Glo3/7 Assay. Caspase activities are normalized to the non-treated cells and presented in relative units. Mean and standard deviations are shown (*n* = 3). Statistical analysis was performed by ANOVA test (upper lane) or by paired Students *t*-test (angular area). **(C)** MDA-MB-231 cells were treated with 300 μg/ml RL2, 200 ng/ml TRAIL, or their combination for indicated time points and subjected to Western blot analysis with the indicated antibodies. Caspase-8 cleavage products p43/p41and caspase-3 cleavage products are indicated. One representative Western blot of two independent experiments is shown. Quantification of the protein expression was normalized to GAPDH. Quantification is shown under specific areas. **(D)** MDA-MB 231 cells were treated with 200 μg/ml RL2 (“R”), 100 ng/ml TRAIL (“T”), their combination (“R + T”), or left untreated (“C”) for 1 h. Cells were separated into “Cytoplasm,” “Mitochondria,” and “Nucleus” fractions. Fractions were analyzed by Western blot with indicated antibodies. SOD2, Actin, and EndoG were used as fraction controls. One representative Western blot of three is shown. Quantification of three independent Western blots for this experiment is shown in Supplementary Figure 3. l.e., long exposure; s.e., short exposure.

Caspase-8 triggers the effector caspase cascade. Accordingly, next, we analyzed whether RL2 treatment impinges on TRAIL-induced effector caspase activity. To this point, caspase-3/7 activation in MDA-MB-231 cells upon RL2, TRAIL, or their co-treatment was investigated. In concordance with the results of cell viability and caspase-8 activity assays, TRAIL treatment alone induced strong caspase-3/7 activity after stimulation for 3 h. This effector caspase activity was inhibited by RL2 co-treatment (Figure 3A). The same inhibitory effects of RL2 on TRAIL-induced caspase-3/7 activity were observed within 2 h after co-stimulation (Figure 3B). Interestingly, the pretreatment of MDA-MB-231 cells with RL2 for 2 h did not modulate its inhibitory action on TRAIL-induced caspase activity (Figure 3B). These findings were supported by a Western blot analysis of procaspase-3 processing, which demonstrated that RL2 co-treatment inhibits TRAIL-induced procaspase-3 processing (Figure 3C). In particular, downregulation of procaspase-3 and subsequently of its active subunits was observed. Hence, it might be concluded that RL2 inhibits TRAIL-induced effector caspase activation.

Our previous reports have demonstrated that RL2 is targeted to mitochondria, where it interacts with TOM70 (Richter et al., 2020). To check whether there is any alteration in mitochondrial localization of RL2 after co-stimulation with TRAIL, a cellular fractionation was carried out. In line with our previous reports, RL2 was found largely in the mitochondrial fraction under both stimulation conditions: upon single treatment and upon RL2/TRAIL co-treatment (Figure 3D). This allowed us to conclude that RL2 is also targeted to mitochondria upon RL2/TRAIL co-treatment and might act in a similar fashion as upon single treatment.

Activation of effector caspases leads to the cleavage of their substrates, which is leading to the demolition of the cells. One of the caspase substrates, poly (adenosine diphosphate ribose) polymerase 1 (PARP1), has been described to serve as a key marker of effector caspase activation. The analysis of PARP1 cleavage in the nuclear fraction upon RL2/TRAIL co-treatment for 1 h demonstrated that this process is strongly inhibited by RL2 compared with single TRAIL treatment for 1 h (Figure 3D and Supplementary Figure 3). This further supports the inhibitory role of RL2 in TRAIL-induced apoptosis upon short-term treatment. Collectively, these findings indicate that RL2 impairs TRAIL-induced caspase activation at the level of both initiator and effector caspases.

### RL2 Induces Mitophagy and Down-Modulation of Key Apoptotic Proteins

**Figure 4:**
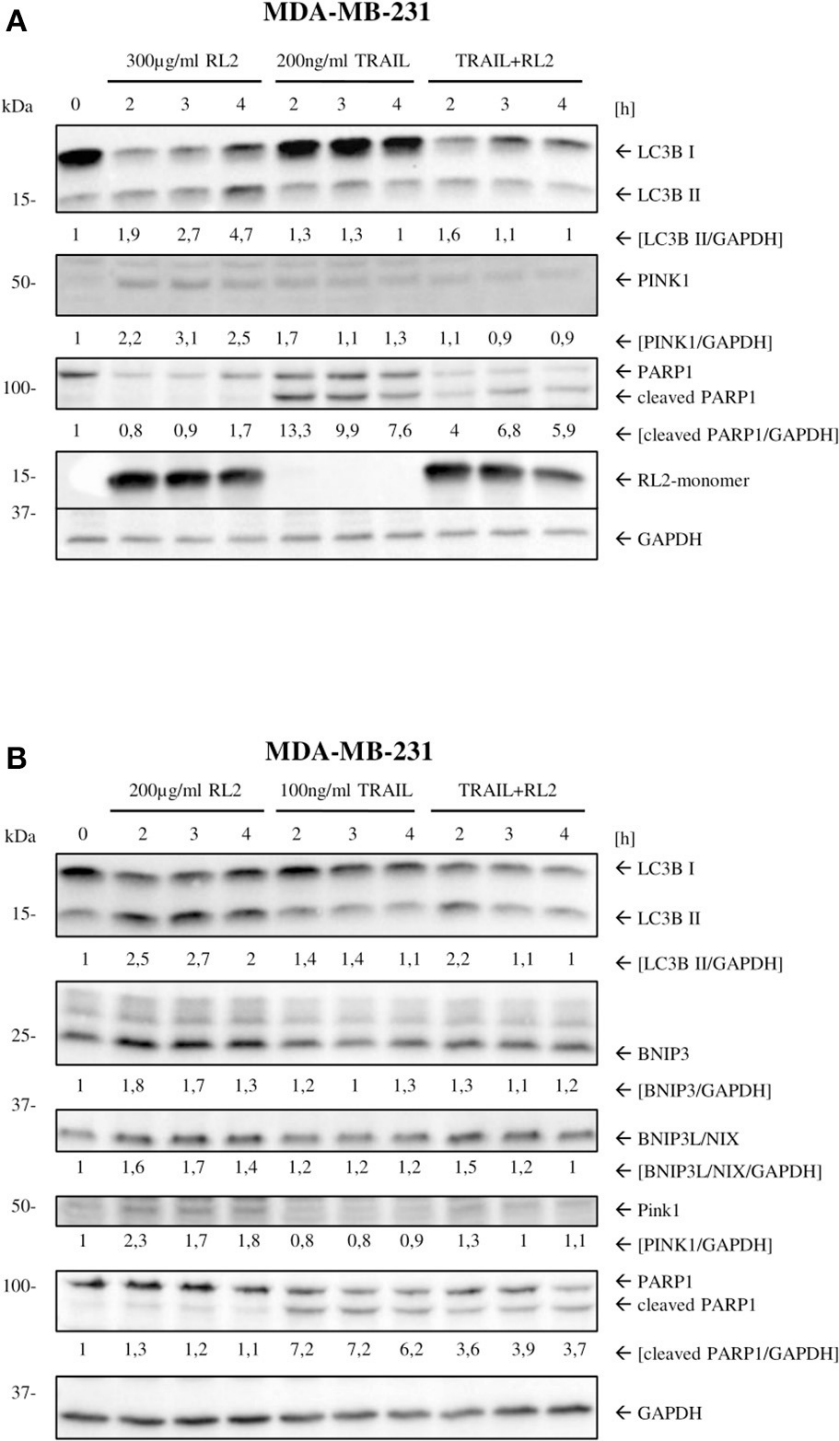
RL2 induces mitophagy during first hours after stimulation. **(A)** MDA-MB-231 cells were treated with 300 μg/ml RL2, 200 ng/ml TRAIL, or the combination of both for indicated time intervals and subjected to Western blot analysis with the indicated antibodies. One representative Western blot of three independent experiments is shown. **(B)** MDA-MB-231 cells were treated with 200 μg/ml RL2 for indicated time intervals and subjected to Western blot analysis with the indicated antibodies. One representative Western blot of three independent experiments is shown. Quantification of three independent Western blots for this experiment is shown in Supplementary Figure 4. Quantification of the protein expression was normalized to GAPDH.

We have previously shown that RL2 interacts with TOM70 at mitochondria (Richter et al., 2020). Moreover, TOM70 serves as a receptor for phosphatase and tensin homolog-induced kinase 1 (PINK1), which is a key initiator protein of mitophagy (Kato et al., 2013). Mitophagy is a selective form of autophagy that is responsible for the removal of damaged mitochondria (Lazarou et al., 2015). The blockage of PINK1 interactions with TOM complex and TOM70 has been reported to induce mitophagy due to the impaired mitochondrial import of PINK1 (Youle and Narendra, 2011). Accordingly, we suggested that RL2 interaction with TOM70 might impair PINK1 import and lead to the induction of mitophagy, which is naturally accompanied by a loss of ATP and mitochondrial membrane potential (Youle and Narendra, 2011; Hamacher-Brady and Brady, 2016). To test this hypothesis, we analyzed the expression of key mitophagy markers (Hamacher-Brady and Brady, 2016) in MDA-MB-231 cells upon RL2 and RL2/TRAIL treatment using Western blot. Already 2 h after RL2 treatment, an increased signal of LC3B-II was detected along with the upregulation of BNIP3, BNIP3L, and PINK1 (Figures 4A,B; Supplementary Figures 4A,B). The same pattern was observed in co-stimulatory treatment with RL2/TRAIL (Figures 4A,B; Supplementary Figures 4A,B). Interestingly, the peak increase in mitophagy markers was observed after approximately 2 h of RL2 treatment, followed by their slow decrease. Moreover, this decrease was more prominent upon co-stimulatory treatment with RL2/TRAIL. The down-modulation of mitophagy markers fits well with the degradation of RL2 in MDA-MB-231 cells (Figures 4A,B). Importantly, upon TRAIL treatment, no increase in LC3B-II was detected, and the upregulation of BNIP3, BNIP3L, and PINK1 was not observed either. Thus, it might be concluded that both RL2 and RL2/TRAIL co-treatment induced mitophagy in MDA-MB-231 cells during short-term treatment.

**Figure 5:**
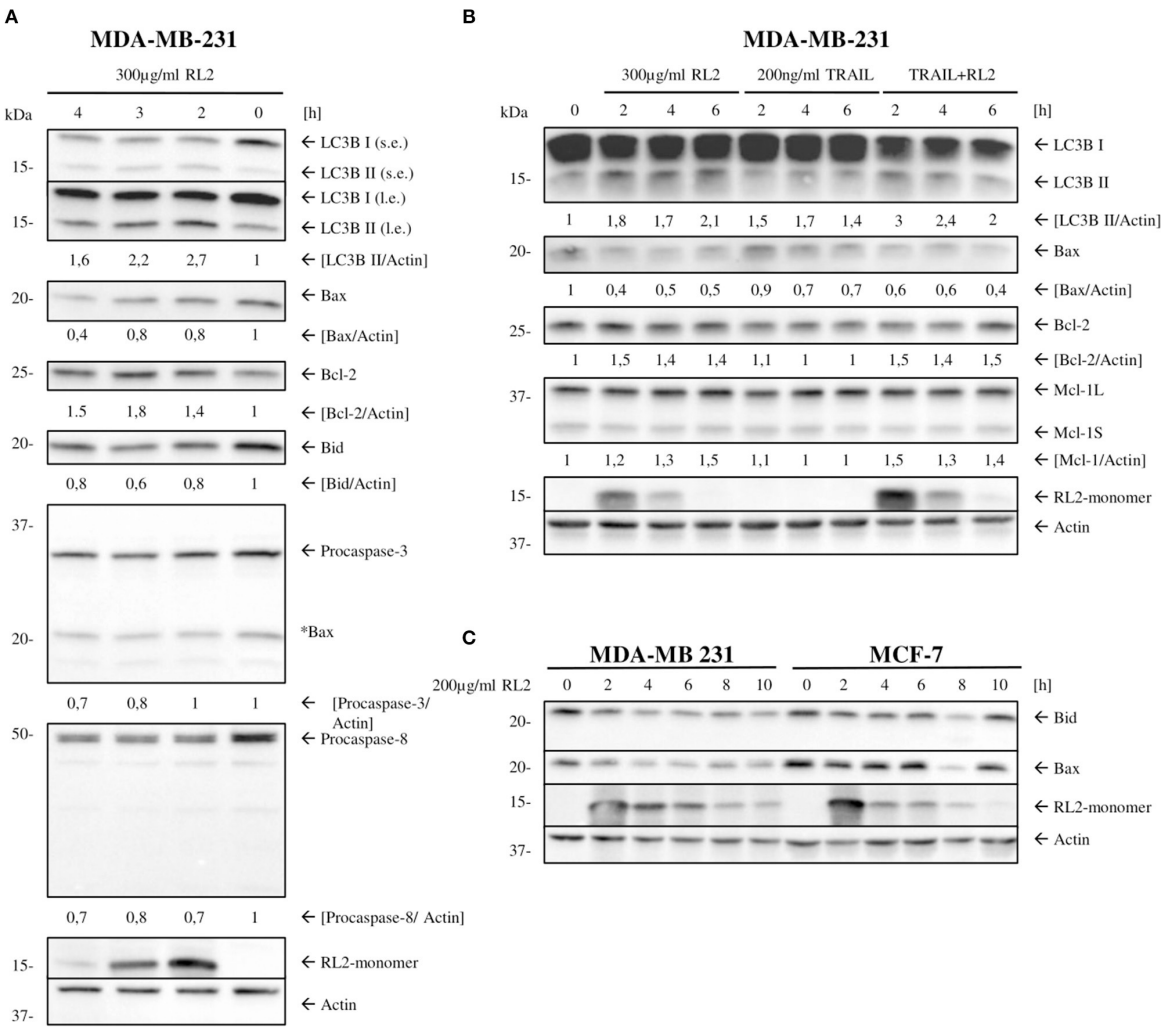
RL2 changes the ratio between antiapoptotic vs. proapoptotic Bcl-2 protein family members. **(A,B**) MDA-MB-231 cells were treated with 300 μg/ml RL2, 200 ng/ml TRAIL, or the combination of both for indicated time intervals and subjected to Western blot analysis with the indicated antibodies. One representative Western blot of three independent experiments is shown. Quantification of the protein expression was normalized to actin. Quantification is shown under specific areas. **(C)** MDA-MB-231 and MCF-7 cells were treated with 200 μg/ml RL2 for indicated time intervals and subjected to Western blot analysis with the indicated antibodies. One representative Western blot of two independent experiments is shown.

Next, we checked whether mitophagy induction by RL2 leads to the down-modulation of core proapoptotic regulators in MDA-MB-231 cells. MDA-MB-231 are classified as type II cells (Aldridge et al., 2011). Therefore, they are dependent on caspase-8-mediated Bid cleavage and tBid generation for the propagation of TRAIL-induced apoptosis. Subsequently, the expression of procaspase-8 and Bcl-2 family members in parallel to LC3 processing was analyzed by Western blot (Figure 5A). RL2 treatment led to the downregulation of proapoptotic Bcl-2 family members Bid and Bax (Figure 5A). In accordance with (Figures 2B,C), procaspase-8a/b down-modulation was observed upon RL2 treatment also in these experiments (Figure 5A). These processes took place strictly in parallel to LC3 processing (Figure 5A). Importantly, the same effects were observed upon RL2/TRAIL co-stimulation (Figure 5B). Indeed, down-modulation of procaspase-8 (Figures 2B,C) and Bax along with LC3 processing (Figure 5B) were monitored upon RL2/TRAIL co-treatment. This suggests that mitophagy-mediated downregulation of key apoptotic proteins blocks TRAIL signaling (Figure 5B). In particular, downregulation of Bid, which serves as a key link between activation of procaspase-8 at the DISC and amplification of the apoptotic signal in mitochondria of type II cells, might significantly contribute to the inhibition of TRAIL-induced apoptosis by RL2 co-treatment. Furthermore, a slight upregulation of Bcl-2 has been observed upon both RL2 and RL2/TRAIL co-treatment (Figure 5). Bcl-2 is a master regulator of life/death decision in type II cells (Scaffidi et al., 1998), and its upregulation might also contribute to the inhibitory effects of RL2 on TRAIL-induced apoptosis in MDA-MB-231 cells. Hence, this analysis demonstrates that the short-term RL2 treatment leads to downregulation of the core apoptotic regulators, including Bax and Bid, procaspases-8 and−3, and upregulation of antiapoptotic Bcl-2 protein and, accordingly, provides the basis for a diminished TRAIL signaling upon RL2/TRAIL stimulation.

Next, we checked whether the effect of RL2 on the expression of Bcl-2 family members could also be observed in other cell lines. The downregulation of Bax and Bid was also uncovered in MCF-7 cells; however, this downregulation was not as strong as in MDA-MB-231 cells, which is in line with the different sensitivity of these two cell lines toward RL2 treatment (Richter et al., 2020) (Figure 5C). These results suggest that RL2 treatment down modulates the key proapoptotic regulators of TRAIL-mediated apoptosis in breast cancer cells, possibly via mitophagy induction.

### RL2/Tumor Necrosis Factor-Related Apoptosis-Inducing Ligand Co-treatment Inhibits Tumor Necrosis Factor-Related Apoptosis-Inducing Ligand-Mediated Cell Death Upon Short Term and Promotes Upon Long-Term Stimulation

**Figure 6:**
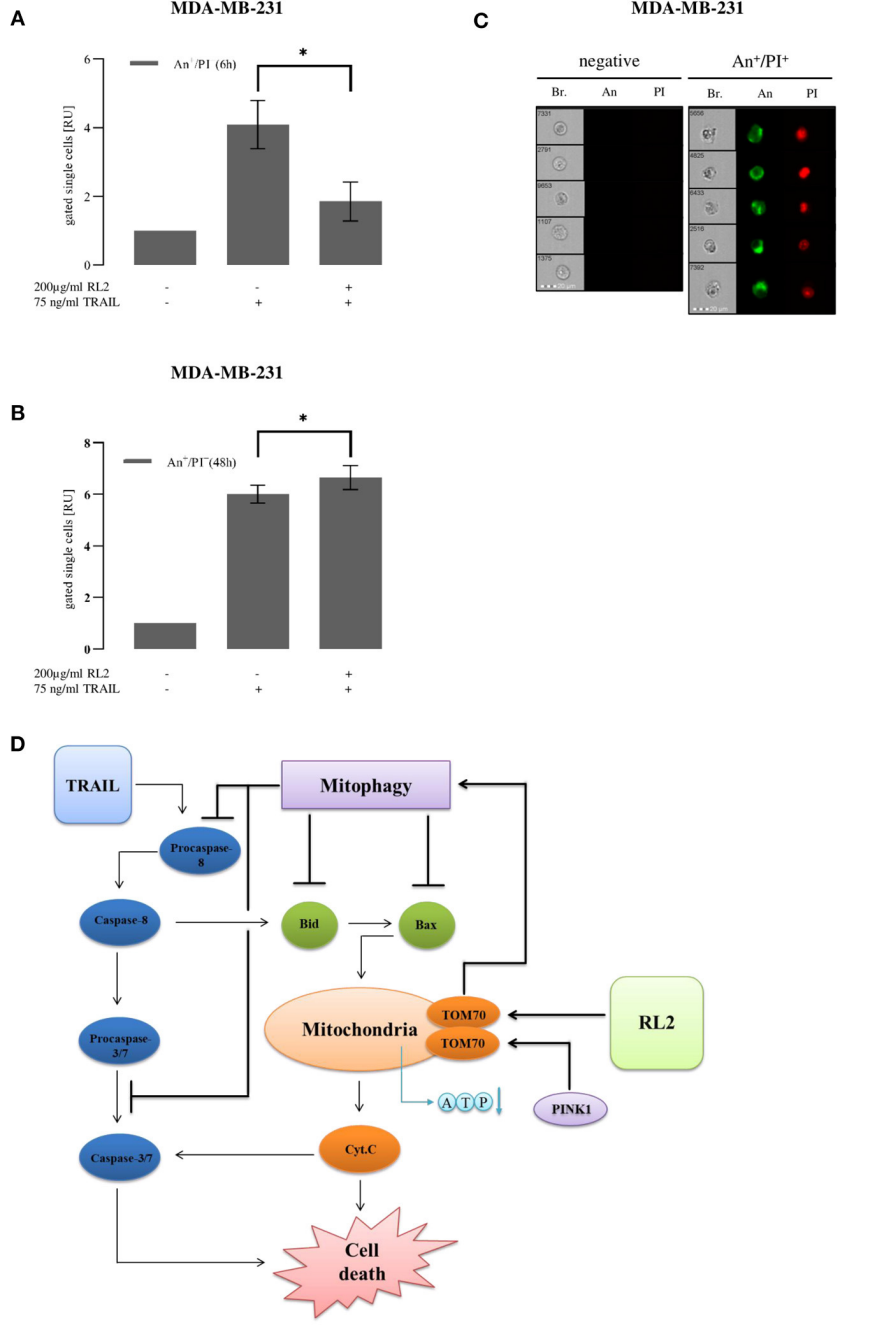
RL2 decreases TRAIL-induced cell death in first hours after TRAIL stimulation and sensitizes cells in long-term treatments. MDA-MB-231 cells were stimulated with indicated concentrations of TRAIL or RL2/TRAIL combination for 6 h **(A)** or 48 h **(B)**. Cell death was measured using annexin V (An)/propidium iodide (PI) staining and analyzed with FlowSight. Amount of double-positive An^+^/PI^+^ cells calculated from three independent experiments are shown in relative units. Statistical analysis was performed by paired Student's *t*-test **(A,B)**. **(C)** Five representative images of double-negative An^−^/PI^−^ (viable) and double-positive An^+^/PI^+^ cells are shown. Abbreviations: Brightfield (Br.), annexin V (An), and propidium iodide (PI). **(D)** Cross talk of RL2- and TRAIL-induced mitophagy and caspase-8 apoptotic cascade.

RL2 co-treatment led to mitophagy induction upon short-term stimulation. To analyze RL2 effects on apoptotic cell death induced by TRAIL in MDA-MB-231 cells upon both short-term and long-term treatments, imaging flow cytometry and propidium iodide/annexin V staining were implemented (Figures 6A–C). TRAIL alone caused a strong increase in the amount of double-positive cells, which was observed already 6 h after TRAIL administration (Figure 6A). This indicates the induction of apoptosis, which was also consistent with the morphology of the TRAIL-treated cells (Figure 6C) (Pietkiewicz et al., 2015). In particular, the formation of apoptotic blebs was observed and typical nuclear changes occurring upon apoptosis induction (Pietkiewicz et al., 2015). The coadministration of RL2 inhibited TRAIL-mediated apoptosis in MDA-MB-231 cells upon short-term treatment (Figure 6A). This was in accordance with data on the inhibitory effects of RL2 on cell viability loss and caspase activity in MDA-MB-231 cells shortly after RL2/TRAIL co-stimulation. Moreover, in line with the observed effects of RL2 on cell viability loss upon the long-term treatment, RL2/TRAIL co-treatment for 48 h resulted in a higher number of dying cells compared with TRAIL-only treatment (Figure 6B). Consistent with the lower sensitivity of MCF-7 cells toward RL2 treatment (Richter et al., 2020), the inhibitory effects of RL2 on apoptosis in MCF-7 cells were observed at later time points (Supplementary Figures 5A,B). Taken together, RL2 inhibited TRAIL-induced apoptosis in MDA-MB-231 and MCF-7 cells upon short-term co-treatment and enhanced cell death upon long-term co-treatment.

## Discussion

Cross talk between different cell death modalities plays a key role in life/death decisions in the cell. Furthermore, the outcome of anticancer therapies strongly depends on the efficiency of cell death induction and the molecular balance of different cell death programs. Importantly, the outcome of combinatory effects upon treatment with several cell death stimuli is essential for the development of efficient anticancer therapies. Here, we addressed the interplay of cell death programs upon coadministration of TRAIL and RL2 to breast cancer cells. Both agents are considered promising cell death inducers in breast cancer cells, and, therefore, their combination is deemed highly important due to its putative therapeutic applications.

We have analyzed two major pathways involved in cell death regulation upon RL2 and TRAIL coadministration: mitophagy and apoptosis. TRAIL induces apoptosis, whereas RL2 induces mitophagy. Mitophagy results in the cellular ATP loss and downregulation of key proapoptotic proteins, which, in turn, leads to apoptosis inhibition. Moreover, two phases appear to be characteristic of the interplay between mitophagy and apoptosis. Initially, RL2-mediated mitophagy blocks apoptosis induction via downregulation of the core proapoptotic regulators, whereas at the later stages, apoptosis takes over. The latter might be connected to the proteolytic degradation of RL2 that was observed in this study and previous reports (Richter et al., 2020). Moreover, the ATP loss induced by mitophagy might lead to the ATP loss mediated necrosis on the single-cell level, which is likely contributing to the overall increase in the number of dying cells upon long-term RL2/TRAIL co-treatment. It has to be mentioned that autophagy induction by RL2 administration has been reported before (Bagamanshina et al., 2019). However, the recent attention toward the action of RL2 at mitochondria (Richter et al., 2020) allowed to suggest in the current study the contribution of mitophagy to RL2-mediated cell death. Moreover, the data presented in this study suggest that the autophagy markers, which were detected previously upon addition of RL2 (Bagamanshina et al., 2019), correspond to mitophagy induction. Hence, our current findings seem to be in accordance with previous reports.

Importantly, our study for the first time decoded the molecular mechanism of the RL2-mediated cell death and demonstrated that RL2 induces mitophagy. This allows solving the contradictions in the literature with respect to the possible mechanisms of RL2-mediated cell death. Furthermore, the interactome analysis of RL2, which deciphered the proteins of the TOM complex and, in particular, TOM70 as a key interaction partner of RL2, serves as important evidence hinting at RL2-induced mitophagy (Richter et al., 2020). TOM70 serves as a receptor for PINK1, which is a key inducer of mitophagy (Kato et al., 2013). The blockage of PINK1 interactions with TOM complex and TOM70 has been reported to induce mitophagy due to the impaired mitochondrial import of PINK1 (Youle and Narendra, 2011). As a result, PINK1 is not processed by PARL within mitochondria and accumulated at the outer mitochondrial membrane, leading to the recruitment of PARKIN that triggers mitophagy (Lazarou et al., 2015). Subsequently, we suggest that RL2 interaction with TOM70 serves as a blocker of TOM70 and thereby impairs PINK1 import. Hence, the induction of mitophagy via RL2 also supports the role of TOM70 and the TOM complex in PINK1 mitochondrial import.

Moreover, the induction of mitophagy provides an explanation for the drop of the mitochondrial membrane and potential ATP synthesis, occurring in the course of mitophagy (Youle and Narendra, 2011), that are observed upon addition of RL2 to the cells in this study as well as in previous studies (Richter et al., 2020). It was suggested previously that the ATP loss upon RL2 administration might be resulting from perturbations of the mitochondrial respiratory chain (Richter et al., 2020). To test this hypothesis, we considered that cells with compromised respiration might shift the energy production to anaerobic glycolysis, and therefore, in this study, oxygen consumption was measured. Importantly, we did not find any changes in oxygen consumption between treated and untreated cells, indicating that the ATP drop is not explained by perturbation of the mitochondrial respiratory chain. Hence, we rather suggest that the ATP loss observed upon RL2 treatment is based on mitophagy induction.

Taken together, we suggest the following scheme for the cross talk between RL2 and TRAIL stimulation (Figure 6D). TRAIL stimulation leads to DISC formation, caspase-8 activation, and apoptosis induction. Furthermore, after the penetration into the cells, RL2 is targeting mitochondria, where it binds to TOM70. This interaction blocks PINK1 transport leading to its accumulation at the outer mitochondrial membrane, loss of mitochondrial membrane potential, and induction of mitophagy. Further, PINK1 accumulation and its consequences result in decreased ATP synthesis and down-modulation of several key proapoptotic regulators, in particular, Bax and Bid, which are key players of the proapoptotic pathway. Moreover, the upregulation of Bcl-2 has been observed upon RL2 treatment, which is a key inhibitor of apoptotic response in type II cells (Scaffidi et al., 1998). This, in turn, might contribute to the down-modulation of TRAIL-induced apoptosis. Importantly, the same inhibitory effects on TRAIL signaling were observed on the other type II cells, ovarian carcinoma CAOV-4 cells. This suggests that RL2 might interfere with TRAIL-induced apoptosis in type II cells. However, all these effects are observed only within the first hours after RL2 administration, as, apparently, at the later time points, RL2 is degraded, and mitophagy is stopped, as was observed by the time-dependent decrease of mitophagy markers along with the decrease in RL2 levels.

The reports on the role of FADD and procaspase-8 in mitophagy and autophagy are still rather incoherent. In the current study, we have observed the downregulation of these two DED proteins leading to the decrease of TRAIL DISC formation and caspase-8 activation (Figure 2). Interestingly, in the previous reports, autophagy has been reported both to promote caspase-8 activity (Laussmann et al., 2011) as well as to down modulate the active caspase-8 (Hou et al., 2010). Moreover, FADD and procaspase-8 were reported to play a suppressor role in autophagy in proliferating T cells (Bell et al., 2008). Further, the formation of iDISC comprising FADD and procaspase-8, leading to caspase-8 activation at the autophagosomal membrane, was reported and interactions of FADD with ATG5 (Gordy and He, 2012). This shows that likely the effects of FADD and procaspase-8 on the mitophagy machinery might be cell-type and time-dependent, and further insights into these processes are required in future studies.

Importantly, upon long-term co-treatment, additive effects of TRAIL and RL2 administration were detected that led to the enhanced loss of cell viability and cell death. As discussed earlier, we suggest that this takes place in the second phase of RL2/TRAIL co-treatment when the effects of mitophagy are down-modulated. The detailed molecular mechanism of combinatorial effects of RL2 and TRAIL has to be addressed in future studies. These pathways might involve the Bcl-2-mediated down-modulation of mitophagy or mitophagy-induced priming of procaspase-8 activity at the later time points similar to the mechanisms mentioned earlier (Laussmann et al., 2011; Hamacher-Brady and Brady, 2016). Our findings demonstrate the potential of the RL2/TRAIL combination for therapeutic applications and the development of new anticancer therapeutic approaches. Moreover, the deregulation of mitophagy has been reported to be a feature of breast cancer cells leading to their resistant phenotype (Bernardini et al., 2017). In particular, the ablation of PINK1/Parkin-mediated mitophagy was reported to regulate the progression of breast cancers. Hence, the possibility of targeting breast cancer cells via administration of RL2 opens new therapeutic opportunities for breast cancer treatment. Moreover, the development of combinatorial treatments on the basis of RL2 might present a perspective approach for other therapeutic strategies for cancers associated with defects in mitophagy.

Taken together, we have further identified the molecular mechanisms of RL2 action in breast cancer cells. Our findings uncovered the molecular details of the cross talk between RL2-induced mitophagy and TRAIL-initiated apoptosis. We have identified that the interplay between mitophagy and TRAIL signaling inhibits cell death in the first stage and promotes it in the second stage. We conclude that the presented findings are likely to have important implications for cancer therapy and might pave the way toward the development of new therapeutic approaches for treating breast cancer.

## Materials and Methods

### Cell Culture

Human adenocarcinoma cells MDA-MB-231 (#ACC 732, DSMZ, Germany) were maintained in Leibovitz L15 media (Gibco™), supplemented with 10% heat-inactivated fetal calf serum, 1% penicillin–streptomycin. Human adenocarcinoma cells MCF-7 (#ACC 115, DSMZ, Germany) were maintained in Roswell Park Memorial Institute 1640 medium (Thermo Fisher Scientific Inc., USA), supplemented with 10% heat-inactivated fetal calf serum, 1% penicillin–streptomycin, 1-mM sodium pyruvate, and 1 × minimum essential medium nonessential amino acids in 5% carbon dioxide (CO_2_). Human ovarian cancer cells CAOV-4, which is a kind gift of Prof. Zhivotovsky (Karolinska Institute, Stockholm, Sweden) (Zamaraev et al., 2018), were maintained in Dulbecco's modified Eagle medium/Ham's F-12 media (Pan-Biotech GmbH, Germany), supplemented with 10% heat-inactivated fetal calf serum, 1% penicillin–streptomycin, and 0.0001% puromycin in 5% CO_2._

### Cell Viability Measurements by Adenosine Triphosphate Assay

MDA-MB-231 (1.2 × 10^4^) or MCF-7 (2 × 10^5^) cells were seeded in 96-well plates. Cells were stimulated in a volume of 50 μl. Measurements were performed according to the manufacturer's instructions (CellTiter-Glo® Luminescent Cell Viability Assay, Promega, Germany) with the addition of 50-μl CellTiter-Glo® solution to each well. The luminescence intensity was analyzed in duplicates using the microplate reader Infinite M200pro (Tecan, Switzerland). The values were normalized against the viability of untreated cells set as one relative unit (RU).

### Cell Viability Measurements by Metabolic Assay

MDA-MB-231 (1.2 × 10^4^) cells were seeded in 96-well plates. Cells were stimulated in a volume of 100 μl (50-μl medium + 50-μl metabolic substrate). Measurements were performed according to the instructions (RealTime-Glo™ MT cell Viability Assay, Promega Germany) with the addition of 50-μl metabolic substrate (2% MT cell viability substrate and 2% NanoLuc™Enzyme) to each well, directly before stimulation. The luminescence intensity was analyzed in duplicates using microplate reader Infinite M200pro (Tecan, Switzerland). The values were normalized against the viability of untreated cells that were set as one RU.

### Caspase-3/7 Activity Assay

MDA-MB-231 (1.2 × 10^4^) cells were seeded in 96-well plates. Cells were stimulated in a volume of 50 μl. Measurements were performed according to the manufacturer's instructions (Caspase-Glo® 3/7 Assay, Promega, Germany) with the addition 50 μl of the Caspase-Glo®3/7 solution to each well. The luminescence intensity was analyzed in duplicates by the microplate reader Infinite M200pro (Tecan, Switzerland). The values were normalized against caspase activity of non-treated cells and set as one RU.

### Caspase-8 Activity Assay

MDA-MB-231 (1.2 × 10^4^) cells were seeded in 96-well plates. Cells were stimulated with the indicated treatments in a volume of 50 μl. After 3 h incubation at 37°C and 5% CO_2_, samples were measured according to the manufacturer's instructions (Caspase-Glo® 8 Assay, Promega, Germany) with the addition 50 μl of the Caspase-Glo®8 (with 0.3% MG-132-Inhibitor) solution to each well. The luminescence intensity was analyzed in duplicates by the microplate reader Infinite M200pro (Tecan, Switzerland). The values were normalized against caspase activity of non-treated cells and set as one RU.

### Oxygen Consumption Rate Measurements

MDA-MB-231 (2.5 × 10^6^) cells were seeded in 10-cm plates. Cells were stimulated with RL2 in a volume of 3 ml for 8 h. Subsequently, cells were trypsinated and resuspended in 500-μl fresh medium. Oxygen consumption rate (OCR) was measured by a Clark-type electrode in the Oxytherm System (Hansatech Instruments Ltd, Norfolk, UK). All samples were measured at 37°C and mixed continuously for ~15 min. The baseline was recorded with 1 ml of fresh medium. Upon stable baseline (OCR < 0.4 nmol × min^−1^ × ml^−1^), the cell samples were measured. Obtained data were fitted to linear regression, and the OCR was expressed in nmol O_2_ × min^−1^ × ml^−1^.

### Cell Death Measurements by Imaging Flow Cytometry

Analysis of cell death induction was performed with FlowSight® Imaging Flow Cytometer (Amnis/MerckMillipore, USA). MDA-MB-231 cells were treated with RL2. Samples were stained with annexin V-fluorescein isothiocyante and propidium iodide. Data were analyzed with IDEAS software version 6.2 (Amnis/MerckMillipore, Darmstadt, Germany), as described previously (Pietkiewicz et al., 2015).

### Western Blot Analysis and Co-immunoprecipitation

Cells (1.25–2.5 × 10^5^) were seeded in six-well plates. Cells were harvested, washed with phosphate-buffered saline (PBS), and lysed for 30 min on ice in lysis buffer (20-mM Tris-hydrochloride, pH 7.4, 137-mM sodium chloride, 2-mM ethylenediaminetetraacetic acid (EDTA), 10% glycerine, 1% Triton X-100, protease inhibitor mix (Roche, Mannheim, Germany) and subjected to Western blot analysis. Sodium dodecyl sulfate-polyacrylamide gel electrophoresis was performed with 12% sodium dodecyl sulfate gels. The TransBlot Turbo system (Biorad, Hercules, USA) was used to blot the gels to nitrocellulose membranes. The membranes were blocked with 5% nonfat dried milk in PBS with 0.05% Tween 20 for 1 h. Washing steps were performed with PBS-Tween three-fold for 5 min. Incubation with primary antibodies was performed overnight at 4°C in PBS-T. HRP-coupled isotype-specific secondary antibodies were incubated for 1 h at room temperature in 5% nonfat dried milk (SantaCruz, Dallas, USA). Chemiluminescence signal was produced with LuminataForte (MerckMillipore, Darmstadt, Germany) and detected with a ChemiDoc imaging system (Biorad, Hercules, USA). Caspase-8 and FADD co-IPs were performed with MDA-MB-231 cells upon RL2, RL2/TRAIL, or TRAIL stimulation. Stimulation was stopped by adding 10-ml cold PBS. Cells were centrifuged for 5 min at 500 × *g* and washed once with cold PBS. Cells were lysed in 500-μl lysis buffer for 30 min on ice and subsequently centrifuged for 15 min at 14,600 × *g*. Fifty-microliter supernatant was used as input control. The remaining supernatant of all samples was adjusted to the same protein concentration and used for immunoprecipitation. Five-microgram FADD or caspase-8 antibody was added to the lysate and incubated for 12 h at 4°C. Samples were subjected to Western blot analysis. DISC co-IP was performed with MDA-MB-231 cells after 1 h of TRAIL or RL2/TRAIL stimulation and via anti-6 x His-tag antibody directed against TRAIL (KillerTRAIL, Enzo Life Sciences). The lysis and co-IP steps were the same as for FADD- and caspase-8-co-IPs. Five-microgram anti-His-Tag antibody was used for each DISC co-IP.

### Cellular Fractionation

MDA-MB-231 cells (2.5 × 10^6^) were stimulated with 100 μg/ml RL2, 50 ng/ml TRAIL, TRAIL/RL2, or left untreated. All centrifugation steps in the following fractionation were performed at 17,000 × *g*. A swelling step was performed in swelling buffer [10-mM 4-(2-hydroxyethyl)-1-piperazineethanesulfonic acid, pH 7.6, 10-mM potassium chloride, 2-mM magnesium chloride, 0.1-mM EDTA, protease Inhibitor mix] for 5 min followed by addition of 0.3% NP-40 (Thermo Fisher Scientific Inc., USA) for 1 min. Centrifugation for 1 min resulted in a separation of the cytoplasmic fraction. The remaining pellet was resuspended in 500-μl swelling buffer and centrifuged for 15 s. The pellet was incubated for 30 min with 40-μl “nucleus buffer” [50-mM 4-(2-hydroxyethyl)-1-piperazineethanesulfonic acid, pH 7.8, 50-mM potassium chloride, 300-mM sodium chloride, 0.1-mM EDTA, 10% glycerol, protease Inhibitor mix], resuspended every 10 min, and centrifuged for 5 min. The nuclei-containing supernatant and mitochondria-containing pellet were separated and the pellet washed twice with PBS. The samples were subjected to Western blot analysis.

### Statistics

Statistical analyses were performed with GraphPad Prism (Version 8.3.0). Paired Student *t*-test and one-way ANOVA test were performed as shown. *p*-Values are based on the following pattern: ns (not significant; *p* > 0.05), ^*^ (significant; *p* < 0.05), ** (significant; *p* < 0.01), *** (significant; *p* < 0.005), and **** (significant; *p* < 0.001).

### Antibodies and Reagents

All chemicals were of analytical grade and purchased from AppliChem (Darmstadt, Germany), CarlRoth (Karlsruhe, Germany), Merck (Darmstadt, Germany), or Sigma-Aldrich (Taufkirchen, Germany). RL2 was purified as described previously (Koval et al., 2014). Z-VAD-FMK (N-1510, Bachem) and recombinant TRAIL (KillerTRAIL, Enzo Life Sciences) were given to cells in indicated concentrations. The following antibodies were used for Western blot analysis: polyclonal anti-Bax antibody (#5023), polyclonal anti-BID antibody (#2002), monoclonal anti-BNIP3 antibody (#44060), monoclonal anti-BNIP3/NIX antibody (#12396), polyclonal anti-caspase-3 antibody (#9662), monoclonal anti-Death Receptor 4 (D9S1T) antibody (#42533), polyclonal anti-endonuclease G (EndoG) antibody (#4969), polyclonal anti-LC3B antibody (#3868), polyclonal anti-PARP1 antibody (#9542), monoclonal anti-PINK1 antibody (#6946), and polyclonal anti-SOD2 antibody (#13194) from Cell Signaling Technology (USA); polyclonal anti-actin antibody (A2103) from Sigma-Aldrich, Germany; monoclonal anti-Bcl-2 antibody (sc-7382), polyclonal anti-GAPDH antibody (sc-48166), and polyclonal anti-Mcl-1 antibody (sc-819) from Santa Cruz Biotechnology (USA); polyclonal anti-κ-Casein antibody (#ab111406), polyclonal anti-6X His Tag antibody (ab9108), and polyclonal anti-TOM70 antibody (ab89624) from Abcam; and monoclonal anti-caspase-8 and anti-FADD antibodies (kindly provided by Prof. P. H. Krammer, DKFZ, Heidelberg). Horseradish peroxidase-conjugated goat anti-mouse immunoglobulins G1 and G2b and goat anti-rabbit and rabbit anti-goat were from Santa Cruz (California, USA).

## Data Availability Statement

The original contributions presented in the study are included in the article/Supplementary Material, further inquiries can be directed to the corresponding author/s.

## Author Contributions

FW, MR, and LO: experiments. FW: manuscript writing. LO, KS, TV-K, EK, VR, and OK: technologies and manuscript writing. IL: manuscript writing and supervision. All authors contributed to the article and approved the submitted version.

## Conflict of Interest

The authors declare that the research was conducted in the absence of any commercial or financial relationships that could be construed as a potential conflict of interest.

## Supplementary Material

Figure S1 RL2 inhibits TRAIL-induced cell viability loss in ovarian carcinoma CAOV-4 cells during the first hours of TRAIL stimulation Caov-4 cells were treated with indicated concentrations of RL2, TRAIL or their combination for 6 h. Cellular ATP levels were measured by using the CellTiter-Glo Luminescent Cell Viability Assay/ CellTiter-Glo Substrate and cell viabilities are normalized to the ones of non-treated cells and presented in relative units (RU). Mean and standard deviations are shown (*n* = 2). The statistical analysis was performed by Anova-test for 6 h.

Figure S2 RL2 has no influence on TRAIL-induced complex II -formation (A, B) MDA-MB 231 cells were treated with the indicated TRAIL, RL2 and RL2/TRAIL concentrations for three hours. Immunoprecipitations (IPs) were done with **(A)** anti-FADD antibodies (FADD IP) and **(B)** anti-Caspase-8 antibodies (Casp.-8-IP). Both IPs were carried out with 5 ìg of the specific antibodies and analysed by Western Blot for the indicated proteins. Bead-control (Beads) were used as control without any antibody addition. One representative experiment out of two is shown for all experiments.

Figure S3 RL2 inhibits TRAIL-induced PARP1 cleavage MDA-MB 231 cells were treated with 200 ìg/ml RL2, 100 ng/ml TRAIL or their combination for 1 h. The cells were separated in ‘Cytoplasm’, ‘Mitochondria’ and ‘Nucleus’ fractions. The fractions were analysed by Western Blot. Bands of cleaved-PARP were quantified against corresponding EndoG bands by ImageLab 5.1beta (Bio-Rad). Three independent Western Blot quantifications are shown.

Figure S4 RL2 treatment induces autophagy/mitophagy **(A)** MDA-MB 231 cells were treated with 300 ìg/ml RL2, 200 ng/ml TRAIL or their combination for indicated time intervals and subjected to Western Blot analysis with the indicated antibodies. **(B)** MDA-MB 231 cells were treated with 200 ìg/ml RL2, 150 ng/ml TRAIL or the combination of both for indicated time intervals and subjected to Western Blot analysis with the indicated antibodies. Quantification of the Western Blot signals was carried out with ImageLab 5.1 beta. Three independent Western Blot quantifications are shown **(A, B)**.

Figure S5 RL2 decreases TRAIL-induced cell death in the first hours after TRAIL stimulation (A,B) MCF-7 cells were stimulated with indicated concentrations of RL2, TRAIL or combination with RL2 for 24 h. Cell death was measured using Annexin V (An) /Propidium Iodide (PI) staining and analysed with FlowSight. **(A)** The amount of An-positive and PI positive cells of three independent experiments is shown in relative units (RU). The statistical analysis was performed by paired Student's t-test. **(B)**. Images of five representative cells for Brightfield (Br.) Annexin V (An) and Propidium Iodide (PI) are shown.

## References

[B1] Aldridge B. B. Gaudet S. Lauffenburger D. A. Sorger P. K. (2011). Lyapunov exponents and phase diagrams reveal multi-factorial control over TRAIL-induced apoptosis. Mol. Syst. Biol. 7:553. 10.1038/msb.2011.85 22108795 PMC3261706

[B2] Bagamanshina A. V. Troitskaya O. S. Nushtaeva A. A. Yunusova A. Y. Starykovych M. O. Kuligina E. V.. (2019). Cytotoxic and antitumor activity of lactaptin in combination with autophagy inducers and inhibitors. Biomed. Res. Int. 2019:4087160. 10.1155/2019/4087160 31317028 PMC6601476

[B3] Bell B. D. Leverrier S. Weist B. M. Newton R. H. Arechiga A. F. Luhrs K. A.. (2008). FADD and caspase-8 control the outcome of autophagic signaling in proliferating T cells. Proc. Natl. Acad. Sci. U. S. A. 105, 16677–16682. 10.1073/pnas.0808597105 18946037 PMC2575479

[B4] Bernardini J. P. Lazarou M. Dewson G. (2017). Parkin and mitophagy in cancer. Oncogene 36, 1315–1327. 10.1038/onc.2016.302 27593930

[B5] Buchbinder J. H. Pischel D. Sundmacher K. Flassig R. J. Lavrik I. N. (2018). Quantitative single cell analysis uncovers the life/death decision in CD95 network. PLoS Comput. Biol. 14:e1006368. 10.1371/journal.pcbi.1006368 30256782 PMC6175528

[B6] Chinak O. A. Shernyukov A. V. Ovcherenko S. S. Sviridov E. A. Golyshev V. M. Fomin A. S.. (2019). Structural and aggregation features of a human kappa-Casein fragment with antitumor and cell-penetrating properties. Molecules 24:2919. 10.3390/molecules24162919 31408975 PMC6721048

[B7] Dickens L. S. Boyd R. S. Jukes-Jones R. Hughes M. A. Robinson G. L. Fairall L.. (2012). A death effector domain chain DISC model reveals a crucial role for caspase-8 chain assembly in mediating apoptotic cell death. Mol. Cell 47, 291–305. 10.1016/j.molcel.2012.05.004 22683266 PMC3477315

[B8] Fu T. M. Li Y. Lu A. Li Z. Vajjhala P. R. Cruz A. C.. (2016). Cryo-EM structure of caspase-8 tandem DED filament reveals assembly and regulation mechanisms of the death-inducing signaling complex. Mol. Cell 64, 236–250. 10.1016/j.molcel.2016.09.009 27746017 PMC5089849

[B9] Galluzzi L. Vitale I. Aaronson S. A. Abrams J. M. Adam D. Agostinis P.. (2018). Molecular mechanisms of cell death: recommendations of the Nomenclature Committee on Cell Death 2018. Cell Death Differ. 25, 486–541. 10.1038/s41418-017-0012-4 29362479 PMC5864239

[B10] Gordy C. He Y. W. (2012). The crosstalk between autophagy and apoptosis: where does this lead? Protein Cell 3, 17–27. 10.1007/s13238-011-1127-x 22314807 PMC4875212

[B11] Hamacher-Brady A. Brady N. R. (2016). Mitophagy programs: mechanisms and physiological implications of mitochondrial targeting by autophagy. Cell Mol. Life Sci. 73, 775–795. 10.1007/s00018-015-2087-8 26611876 PMC4735260

[B12] Hillert L. K. Ivanisenko N. V. Espe J. Konig C. Ivanisenko V. A. Kahne T.. (2020). Long and short isoforms of c-FLIP act as control checkpoints of DED filament assembly. Oncogene 39, 1756–1772. 10.1038/s41388-019-1100-3 31740779

[B13] Hoffmann J. C. Pappa A. Krammer P. H. Lavrik I. N. (2009). A new C-terminal cleavage product of procaspase-8, p30, defines an alternative pathway of procaspase-8 activation. Mol. Cell Biol. 29, 4431–4440. 10.1128/MCB.02261-07 19528225 PMC2725745

[B14] Hou W. Han J. Lu C. Goldstein L. A. Rabinowich H. (2010). Autophagic degradation of active caspase-8: a crosstalk mechanism between autophagy and apoptosis. Autophagy 6, 891–900. 10.4161/auto.6.7.13038 20724831 PMC3039736

[B15] Hughes M. A. Powley I. R. Jukes-Jones R. Horn S. Feoktistova M. Fairall L.. (2016). Co-operative and hierarchical binding of c-FLIP and caspase-8: a unified model defines how c-FLIP isoforms differentially control cell fate. Mol. Cell 61, 834–849. 10.1016/j.molcel.2016.02.023 26990987 PMC4819448

[B16] Kato H. Lu Q. Rapaport D. Kozjak-Pavlovic V. (2013). Tom70 is essential for PINK1 import into mitochondria. PLoS ONE 8:e58435. 10.1371/journal.pone.0058435 23472196 PMC3589387

[B17] Kaufmann T. Strasser A. Jost P. J. (2012). Fas death receptor signalling: roles of Bid and XIAP. Cell Death Differ. 19, 42–50. 10.1038/cdd.2011.121 21959933 PMC3252833

[B18] Koval O. A. Fomin A. S. Kaledin V. I. Semenov D. V. Potapenko M. O. Kuligina E. V.. (2012). A novel pro-apoptotic effector lactaptin inhibits tumor growth in mice models. Biochimie 94, 2467–2474. 10.1016/j.biochi.2012.08.017 22968174

[B19] Koval O. A. Tkachenko A. V. Fomin A. S. Semenov D. V. Nushtaeva A. A. Kuligina E. V.. (2014). Lactaptin induces p53-independent cell death associated with features of apoptosis and autophagy and delays growth of breast cancer cells in mouse xenografts. PLoS ONE 9:e93921. 10.1371/journal.pone.0093921 24710119 PMC3978064

[B20] Krammer P. H. Arnold R. Lavrik I. N. (2007). Life and death in peripheral T cells. Nat. Rev. Immunol. 7, 532–542. 10.1038/nri2115 17589543

[B21] Lafont E. Kantari-Mimoun C. Draber P. De Miguel D. Hartwig T. Reichert M.. (2017). The linear ubiquitin chain assembly complex regulates TRAIL-induced gene activation and cell death. EMBO J. 36, 1147–1166. 10.15252/embj.201695699 28258062 PMC5412822

[B22] Laussmann M. A. Passante E. Dussmann H. Rauen J. A. Wurstle M. L. Delgado M. E.. (2011). Proteasome inhibition can induce an autophagy-dependent apical activation of caspase-8. Cell Death Differ. 18, 1584–1597. 10.1038/cdd.2011.27 21455219 PMC3130899

[B23] Lavrik I. Krueger A. Schmitz I. Baumann S. Weyd H. Krammer P. H.. (2003). The active caspase-8 heterotetramer is formed at the CD95 DISC. Cell Death Differ. 10, 144–145. 10.1038/sj.cdd.4401156 12655304

[B24] Lavrik I. N. Krammer P. H. (2012). Regulation of CD95/Fas signaling at the DISC. Cell Death Differ. 19, 36–41. 10.1038/cdd.2011.155 22075988 PMC3252827

[B25] Lazarou M. Sliter D. A. Kane L. A. Sarraf S. A. Wang C. Burman J. L.. (2015). The ubiquitin kinase PINK1 recruits autophagy receptors to induce mitophagy. Nature 524, 309–314. 10.1038/nature14893 26266977 PMC5018156

[B26] Lemke J. von Karstedt S. Zinngrebe J. Walczak H. (2014). Getting TRAIL back on track for cancer therapy. Cell Death Differ. 21, 1350–1364. 10.1038/cdd.2014.81 24948009 PMC4131183

[B27] Pietkiewicz S. Schmidt J. H. Lavrik I. N. (2015). Quantification of apoptosis and necroptosis at the single cell level by a combination of Imaging flow cytometry with classical annexin V/propidium iodide staining. J. Immunol. Methods 423, 99–103. 10.1016/j.jim.2015.04.025 25975759

[B28] Richter M. Wohlfromm F. Kahne T. Bongartz H. Seyrek K. Kit Y.. (2020). The recombinant fragment of human kappa-casein induces cell death by targeting the proteins of mitochondrial import in breast cancer cells. Cancers 12:1427. 10.3390/cancers12061427 32486420 PMC7352597

[B29] Scaffidi C. Fulda S. Srinivasan A. Friesen C. Li F. Tomaselli K. J.. (1998). Two CD95 (APO-1/Fas) signaling pathways. EMBO J. 17, 1675–1687. 10.1093/emboj/17.6.1675 9501089 PMC1170515

[B30] Schleich K. Warnken U. Fricker N. Ozturk S. Richter P. Kammerer K.. (2012). Stoichiometry of the CD95 death-inducing signaling complex: experimental and modeling evidence for a death effector domain chain model. Mol. Cell 47, 306–319. 10.1016/j.molcel.2012.05.006 22683265

[B31] Semenov D. V. Fomin A. S. Kuligina E. V. Koval O. A. Matveeva V. A. Babkina I. N.. (2010). Recombinant analogs of a novel milk pro-apoptotic peptide, lactaptin, and their effect on cultured human cells. Protein J. 29, 174–180. 10.1007/s10930-010-9237-5 20232123

[B32] Spencer S. L. Gaudet S. Albeck J. G. Burke J. M. Sorger P. K. (2009). Non-genetic origins of cell-to-cell variability in TRAIL-induced apoptosis. Nature 459, 428–432. 10.1038/nature08012 19363473 PMC2858974

[B33] Sprick M. R. Rieser E. Stahl H. Grosse-Wilde A. Weigand M. A. Walczak H. (2002). Caspase-10 is recruited to and activated at the native TRAIL and CD95 death-inducing signalling complexes in a FADD-dependent manner but can not functionally substitute caspase-8. EMBO J. 21, 4520–4530. 10.1093/emboj/cdf441 12198154 PMC126181

[B34] Sprick M. R. Weigand M. A. Rieser E. Rauch C. T. Juo P. Blenis J.. (2000). FADD/MORT1 and caspase-8 are recruited to TRAIL receptors 1 and 2 and are essential for apoptosis mediated by TRAIL receptor 2. Immunity 12, 599–609. 10.1016/S1074-7613(00)80211-3 10894160

[B35] von Karstedt S. Montinaro A. Walczak H. (2017). Exploring the TRAILs less travelled: TRAIL in cancer biology and therapy. Nat. Rev. Cancer 17, 352–366. 10.1038/nrc.2017.28 28536452

[B36] Walczak H. Haas T. L. (2008). Biochemical analysis of the native TRAIL death-inducing signaling complex. Methods Mol. Biol. 414, 221–239. 10.1007/978-1-59745-339-4_16 18175822

[B37] Youle R. J. Narendra D. P. (2011). Mechanisms of mitophagy. Nat. Rev. Mol. Cell Biol. 12, 9–14. 10.1038/nrm3028 21179058 PMC4780047

[B38] Zamaraev A. V. Kopeina G. S. Buchbinder J. H. Zhivotovsky B. Lavrik I. N. (2018). Caspase-2 is a negative regulator of necroptosis. Int. J. Biochem. Cell Biol. 102, 101–108. 10.1016/j.biocel.2018.07.006 30025878

