# Supplementary figures and images for: Interplay Between Mitophagy and Apoptosis Defines a Cell Fate Upon Co-treatment of Breast Cancer Cells With a Recombinant Fragment of Human κ-Casein and Tumor Necrosis Factor-Related Apoptosis-Inducing Ligand
Source: Front Cell Dev Biol. 2021 Jan 18;8:617762. doi: 10.3389/fcell.2020.617762 (PMC7849764; doi:10.3389/fcell.2020.617762)

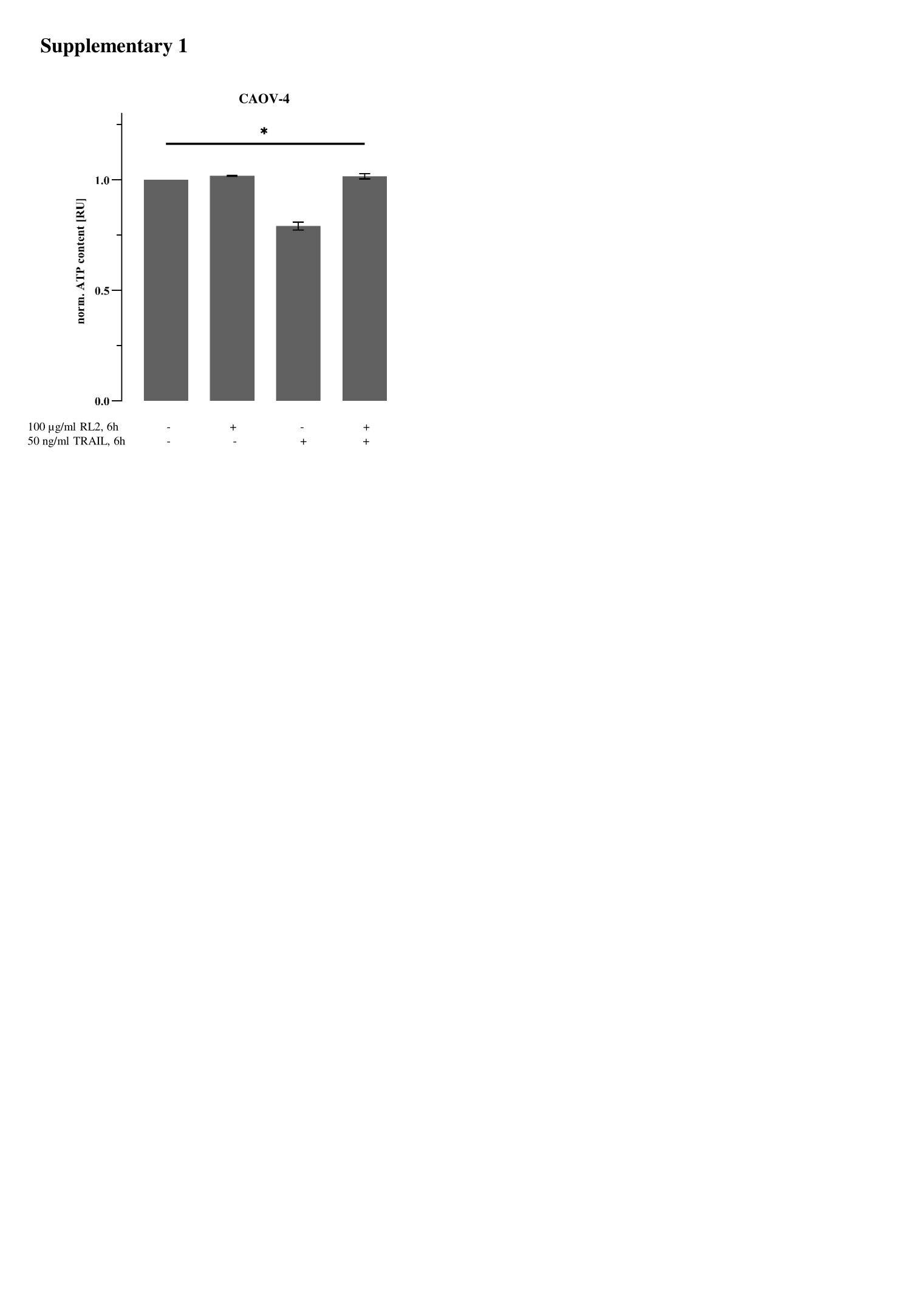

Supplement: Figure S1 — RL2 inhibits TRAIL-induced cell viability loss in ovarian carcinoma CAOV-4 cells during the first hours of TRAIL stimulation Caov-4 cells were treated with indicated concentrations of RL2, TRAIL or their combination for 6 h. Cellular ATP levels were measured by using the CellTiter-Glo Luminescent Cell Viability Assay/ CellTiter-Glo Substrate and cell viabilities are normalized to the ones of non-treated cells and presented in relative units (RU). Mean and standard deviations are shown (n = 2). The statistical analysis was performed by Anova-test for 6 h. [file Image_1.tiff]

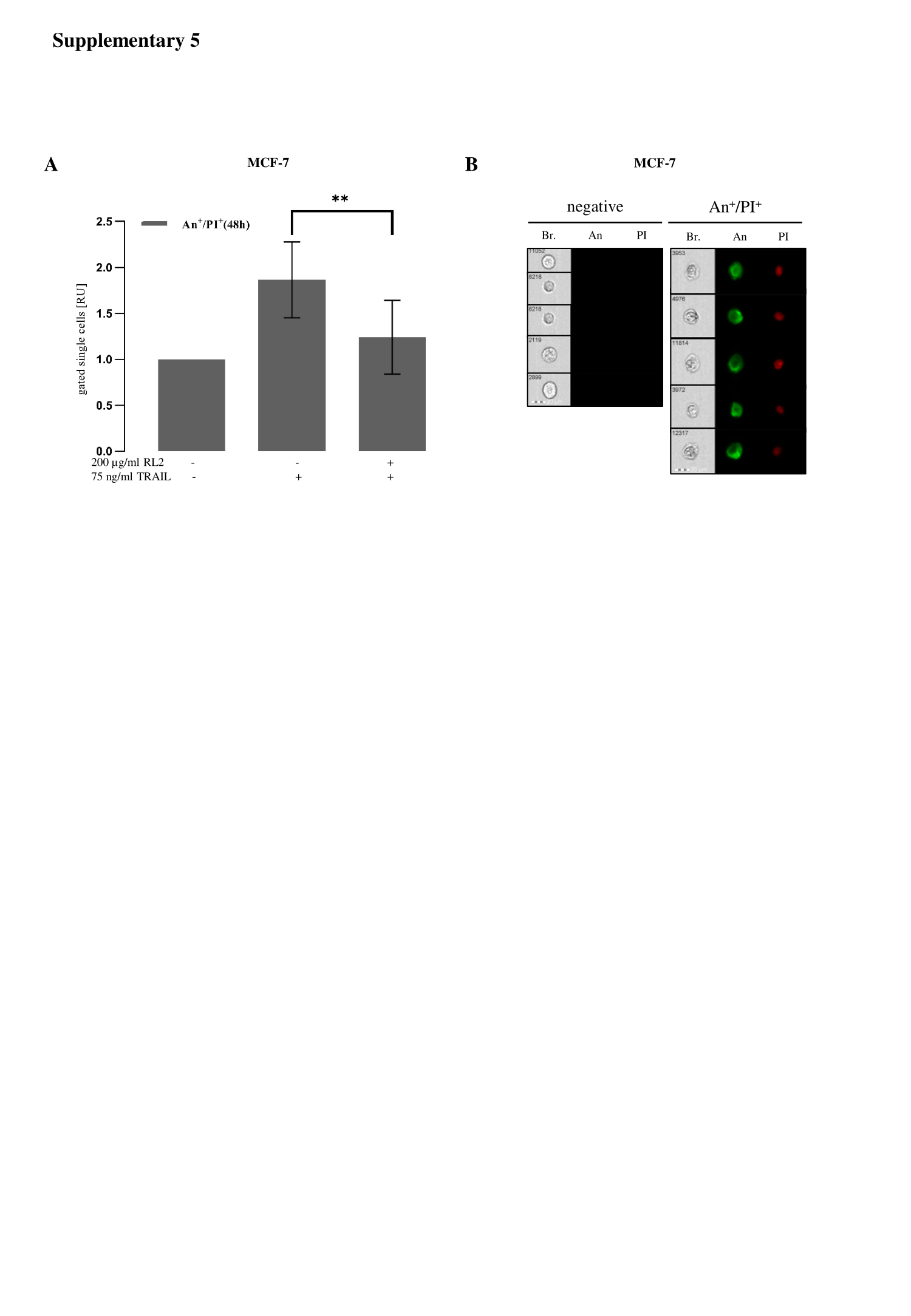

Supplement: Figure S5 — RL2 decreases TRAIL-induced cell death in the first hours after TRAIL stimulation (A,B) MCF-7 cells were stimulated with indicated concentrations of RL2, TRAIL or combination with RL2 for 24 h. Cell death was measured using Annexin V (An) /Propidium Iodide (PI) staining and analysed with FlowSight. (A) The amount of An-positive and PI positive cells of three independent experiments is shown in relative units (RU). The statistical analysis was performed by paired Student's t-test. (B). Images of five representative cells for Brightfield (Br.) Annexin V (An) and Propidium Iodide (PI) are shown. [file Image_5.tiff]
